# Supplementary material for: From promise to practice: pairing non-invasive sampling with genomics in conservation
Source: PeerJ. 2015 Jul 21;3:e1106. doi: 10.7717/peerj.1106 (PMC4517967; doi:10.7717/peerj.1106)
Supplement: Table S1 [file peerj-03-1106-s001.docx]

Table S1. Summarized Structure output including Δ*K* for the Thornton Lake elevational transect based on 37 outlier loci.

| *K* | Reps | Mean ln P (*K*) | Stdev ln P (*K*) | ln' (*K*) | \|ln' '(*K*)\| | Δ*K* |
| --- | --- | --- | --- | --- | --- | --- |
| 1 | 10 | -1524.4 | 0.1912 | n/a | n/a | n/a |
| 2 | 10 | -1246.3 | 0.2111 | 278.1 | 99.9 | 473.4 |
| 3 | 10 | -1068.1 | 0.3802 | 178.2 | 119.6 | 314.6 |
| 4 | 10 | -1009.5 | 0.5673 | 58.6 | 24.8 | 43.7 |
| 5 | 10 | -975.7 | 0.9766 | 33.8 | 38.8 | 39.7 |
| 6 | 10 | -980.6 | 2.2462 | -4.9 | n/a | n/a |
